# Envisioning family, but where? Parenthood-related geographic preferences among childless LGB and heterosexual adults in Ecuador

**DOI:** 10.3389/fsoc.2026.1876346

**Published:** 2026-08-21

**Authors:** Carlos Hermosa-Bosano, Paula Hidalgo-Andrade, Rafael Borja-Moreano, César Parra-Gaete

**Affiliations:** 1 Grupo de Investigación Bienestar, Salud y Sociedad, Escuela de Psicología y Educación, Universidad de Las Américas, Quito, Ecuador; 2 Escuela de Psicología y Educación, Universidad de Las Américas, Quito, Ecuador

**Keywords:** LGB-parenting, parenthood aspirations, parenthood desires, same-sex parenting, sexual migration, geographic preferences

## Abstract

**Introduction:**

Research on parenthood aspirations among lesbian, gay, and bisexual (LGB) adults has expanded, yet little is known about the geographic contexts in which parenthood is envisioned, particularly in the Global South. This question has practical relevance because unequal legal recognition, social acceptance, economic opportunity, and personal safety may make the exercise of family formation rights partly contingent on mobility.

**Methods:**

Using a cross-sectional convergent mixed-methods survey, we examined parenthood-related geographic preferences among 184 childless cisgender adults residing in Ecuador who expressed a desire to become parents (LGB: *n* = 71; heterosexual: *n* = 113). Closed-ended responses were analyzed quantitatively; and open-ended explanations were examined using content analysis.

**Results:**

LGB participants more frequently preferred living abroad than heterosexual participants, Yates-corrected *χ*^2^(1, *N* = 184) = 9.54, *p* = 0.002, Cramér’s *V* = 0.24. In a logistic regression (*n* = 177), heterosexual participants had lower adjusted odds of preferring another country than LGB participants (aOR = 0.26, 95% CI [0.12, 0.56], *p* < 0.001). Open-ended responses showed that better opportunities and quality of life were salient across groups, while a subset of LGB participants additionally referred to restrictive social attitudes or limited recognition of same-gender-parent families. Among participants preferring another country, Spain, the United States, and Canada were the most frequently identified destinations. These preferences reflect anticipated conditions rather than migration plans or behavior.

**Discussion:**

Overall, these findings suggest that material conditions and anticipated social environments shape where prospective parents imagine family formation, with sexuality-related constraints playing a particularly relevant role for some LGB adults in Ecuador. Thus, this study contributes to emerging scholarship by integrating perspectives on family formation and mobility in underexamined Global South settings.

## Introduction

Parenthood is widely understood as a central life goal, motivated by the desire to give and receive love, create a family, and achieve personal fulfillment (Langdridge et al., 2005). Across cultural contexts, it functions as a central marker of adulthood and well-being, reinforced by social norms that promote a traditional sequence of romantic partnership, marriage, and childrearing, particularly in heterosexual relationships (Rimal and Real, 2003; Tate, 2022). These cultural scripts prescribe parenthood as an expected life event, producing pressures that extend into adulthood. Individuals who depart from this trajectory, whether through voluntary childlessness, single parenthood, or same-gender parenting, are often met with stigma or social judgment (Ashburn-Nardo, 2017; Ekelund and Ask, 2021; Stavrova and Fetchenhauer, 2015).

Within this context, a growing body of research shows that many lesbian, gay, and bisexual (LGB) individuals express desires and intentions to become parents, albeit at lower rates than their heterosexual counterparts (Costa and Tasker, 2018; Tate et al., 2019). These differences reflect the complex influence of structural, legal, and social barriers that shape both the feasibility and desirability of parenthood for LGB populations (Gato et al., 2017). In Latin America, emerging studies have begun to document how LGB individuals actively construct pathways to parenthood under constrained conditions, highlighting the role of legal frameworks, socioeconomic resources, and interpersonal support in shaping their aspirations (Araldi and Serralta, 2019).

A crucial yet often overlooked aspect of these aspirations concerns how individuals evaluate the conditions they consider necessary or desirable for becoming parents, including the geographic contexts in which they envision having and raising their children. For some, remaining in their country of residence may be compatible with these aspirations. For others, relocating abroad may emerge as a strategic option to overcome social and structural barriers, including restrictive adoption laws, limited access to reproductive technologies, institutional discrimination, and economic instability (Yue, 2013).

In this sense, migration can be understood as a condition through which some individuals may attempt to align their parenthood aspirations with more favorable structural conditions. This dynamic may be particularly salient in *Global South*^1^ countries such as Ecuador, where, despite some legal advancements in the rights of sexually and gender-diverse populations, significant obstacles to parenthood remain. In such contexts, migration may provide access to more favorable legal frameworks, economic opportunities, and broader social acceptance, thereby enabling individuals to pursue parenthood under more supportive conditions. Despite growing research on both parenthood aspirations and LGB migration, little is known about how these domains intersect when individuals envision family formation, particularly in Global South contexts. To address this gap, this study examined parenthood aspirations and parenthood-related geographic preferences among childless LGB and heterosexual adults residing in Ecuador.

### Parenthood aspirations of LGB individuals

Parenthood aspirations refer to an individual’s expressed desire, intention, or hope to become a parent at some point in their future (Shenkman and Abramovitch, 2021; Shenkman and Shaia, 2022). Scholars typically distinguish between *desires*, understood as the expressed wish to have children, and *intentions*, which involve concrete plans to pursue parenthood (Shenkman and Abramovitch, 2021; Shenkman and Shaia, 2022; Simon et al., 2018). Aspirations are particularly relevant because they capture not only individual preferences but also how individuals interpret and respond to the social structures, opportunities, and constraints that surround them. In this sense, parenthood aspirations are shaped not only by personal motivations, but also by the conditions under which parenthood is perceived as feasible, including social, legal, and material contexts (Tate and Patterson, 2019).

Research consistently shows that LGB individuals report lower levels of both desires and intentions compared to their heterosexual counterparts (Baiocco and Laghi, 2013; Riskind and Patterson, 2010). A variety of demographic factors, including gender, age, education, religion, and racial or ethnic identity, shape how LGB individuals envision and plan for parenthood (Costa and Bidell, 2017; Scandurra et al., 2019; Simon et al., 2018). Psychosocial factors, such as support from partners, family, and broader social networks, also play a critical role (Leal et al., 2019; Salinas-Quiroz et al., 2020). At a structural level, legal obstacles such as adoption bans and ambiguous access to assisted reproductive technologies, alongside persistent institutional heterosexism, can significantly constrain not only the feasibility and accessibility of parenthood for LGB populations, but also its desirability (Bauermeister, 2014; Meslay and Russell, 2025). These constraints may also influence where individuals imagine parenthood as possible, particularly when local conditions limit access to pathways for family formation.

To date, most empirical research on LGB parenthood aspirations has been conducted in the Global North, particularly in the United States (Riskind and Patterson, 2010; Tate, 2022) and Europe (e.g., Portugal, the UK, and Italy; Baiocco and Laghi, 2013; Costa and Bidell, 2017; Shenkman et al., 2021). In the Global South, research has focused mainly on Asian and Middle Eastern contexts (e.g., Taiwan, China, Israel) (Li and Patterson, 2022; Shenkman and Shaia, 2022). These studies have provided valuable insights into how cultural norms, values, and sociopolitical conditions shape LGB individuals’ aspirations and pathways to parenthood.

In Latin America, empirical research remains limited and predominantly qualitative. For instance, in Chile, Herrera et al. (2018) interviewed 14 gay fathers to explore their transition into parenthood and the fulfillment of their parenting desires. The study found that many participants sought to replicate the normative two-parent family model and that higher socioeconomic status facilitated safer and more stable parenting environments through improved access to housing, education, and healthcare. In Brazil, Araldi and Serralta (2019) conducted interviews with same-sex couples and found that parenthood was often pursued after achieving financial and professional stability, with emotional motivations and close family ties playing a central role. These authors also reported that the pathways to parenthood, such as adoption, prior heterosexual relationships, assisted reproduction, or co-parenting, were significantly shaped by legal and cultural barriers. Quantitative research in the region is scarce. One exception is Salinas-Quiroz et al. (2020), who examined parenthood aspirations among sexually and gender-diverse individuals in Mexico. The study found low levels of parenthood desire across the sample, with lower levels reported among plurisexual (i.e., bisexual, pansexual, and queer) and transgender participants. Together, these studies demonstrate that parenthood is embedded in local institutions and inequalities, but they offer limited evidence about its geographic dimension, that is, where prospective parents believe they could establish and raise a family.

For some LGB individuals, parenthood aspirations may not be tied to their country of origin but rather imagined in other national contexts that provide more supportive legal, economic, and sociocultural conditions. In contrast, heterosexual individuals may be more likely to envision parenthood within their home countries, given greater social and legal acceptance. Comparing LGB and heterosexual participants can clarify these dynamics. People across sexual orientation groups may consider economic opportunities, security, family support, and quality of life when evaluating where to raise children. LGB individuals may face these broadly shared concerns while also anticipating sexuality-related barriers, such as discrimination, lack of recognition, or restricted access to family-formation services. The comparison therefore helps identify concerns common to prospective parents as well as concerns potentially patterned by unequal exposure to sexuality-related constraints.

### Migration among LGB individuals

Migration is a multifaceted phenomenon encompassing both voluntary and involuntary movements, temporary and permanent (Ottonelli and Torresi, 2013). It is not merely a personal decision but one shaped by structural forces such as economic inequality, political instability, and environmental crises (Castles et al., 2014). Early migration studies often centered on male, cisgender, heterosexual subjects, marginalizing the experiences of sexually and gender-diverse individuals (Green, 2020). In response, scholars introduced the concept of sexual migration, referring to movements motivated, in whole or in part, by sexuality-related factors (Carrillo, 2004). These motivations may include the pursuit of sexual freedom, romantic relationships, identity exploration, or the desire to escape discrimination and attain greater rights and safety (Carrillo, 2004).

Building on this perspective, migration may function not only as a response to constraint, but also as a means through which individuals seek to realize desired life trajectories. As Manalansan (2006) argues, conventional migration paradigms often reduce non-heterosexual migration to narratives of persecution or economic necessity, whereas sexual migration highlights affective, relational and identity-based motivations. In this context, migration may serve not only as a survival strategy but also to enact alternative futures, including the formation of families.

Although the literature increasingly recognizes the intersection of migration, sexuality, and identity, the specific link between migration and parenthood aspirations remains unexplored. Existing studies have examined how LGB individuals migrate for legal protection, community, or economic opportunity (Hoffmann and Velasco, 2023; Valenzuela, 2021), and how mobility is shaped by intersecting factors such as race, gender, class, and religion (Kalra and Bhugra, 2010). However, to the best of our knowledge, less attention has been paid to how aspirations for parenting and family formation are related. This gap is important, as it limits our understanding of how individuals integrate desires for parenthood with considerations of place and mobility.

In Latin America, research on LGB migration remains limited. Despite recent legal advances, public attitudes remain ambivalent, and discrimination persists (Chaux et al., 2021; Corrales, 2020). While some individuals move within countries toward urban centers, others migrate internationally in search of safety, anonymity, or legal recognition. For example, regional mobility has been described to explore sexuality in more open contexts (Stang Alva, 2019), and countries with more progressive legal frameworks have become destinations for sexually and gender-diverse populations, despite persistent discrimination (Fortes De Lena, 2024; Perez Ripossio, 2021).

Altogether, this emerging body of literature questions traditional views of LGB migration as mainly reactive. It underscores the importance of recognizing affective and aspirational dimensions of mobility, particularly those related to family, kinship, and reproduction. While some studies acknowledge that LGB individuals migrate in search of greater economic stability or safety, fewer have explored how desires for parenting, caregiving, or forming alternative family structures may relate to these movements.

### This study

Ecuador provides an important setting in which to examine these gaps because formal legal advances coexist with continuing institutional and social constraints. Same-sex marriage was legalized in 2019, representing a major expansion of LGBT rights. However, access to some family-formation pathways and the legal recognition of same-gender parenting remains unequal or uncertain, particularly in relation to joint adoption and assisted reproduction (Equaldex, 2024). National evidence has also documented experiences of violence and discrimination among sexually and gender-diverse people in family, educational, healthcare, employment and legal contexts (INEC, 2013). These conditions coexist with broader concerns about employment, economic precarity, security, and quality of life that may affect prospective parents regardless of sexual orientation. These factors may lead some LGB individuals to view migration not only as a route to safety or economic opportunity but also as a strategy for family formation. Migration thus becomes a way to imagine and pursue life trajectories that include parenthood under more favorable conditions.

The present study examined parenthood-related geographic preferences among childless cisgender adults residing in Ecuador who had already expressed a desire to have children. It focused on whether participants preferred to live in their current country of residence or another country when considering future parenthood and on the reasons underlying that preference. The quantitative component assessed whether sexual orientation was associated with geographic preference before and after adjustment for sociodemographic characteristics. The qualitative component examined how respondents explained preferences for the current country or another country and which destinations were identified by those preferring another country.

Thus, this study addressed three research questions:

1. Among childless adults in Ecuador who desire parenthood, is sexual orientation associated with preferring another country rather than the current country of residence when considering future parenthood?
2. Does the association between sexual orientation and parenthood-related geographic preference remain after adjustment for age, gender, education, employment status, and income?
3. What reasons do heterosexual and LGB respondents give for preferring the current country or another country, and which destinations do respondents who prefer another country identify?

Based on evidence that LGB adults may anticipate additional legal, institutional, and social constraints on family formation, we expected LGB participants to have higher odds than heterosexual participants of preferring another country. The qualitative component was exploratory; therefore, no directional hypotheses were specified for the content categories.

## Methods

### Design and participants

This study is part of a broader initiative to explore the parenthood aspirations of childless individuals residing in Ecuador. We conducted a non-experimental, cross-sectional, convergent mixed-methods design and collected data through an online survey. Closed-ended and open-ended responses were collected during the same administration, analyzed separately, and integrated during interpretation.

A total of 561 individuals initially participated. For this article, we focus on a subsample that met the following inclusion criteria: (1) 18 years of age or older, (2) no biological or adoptive children at the time of the study, (3) residing in Ecuador during the data collection period, and (4) self-identifying as cisgender, that is, identifying as a man or woman and not identifying as transgender. We focused on cisgender individuals as not enough participants identified as transgender; we acknowledge that this excludes transgender and nonbinary experiences and believe they should be analyzed in greater depth in future studies.

A total of 367 participants met the broader eligibility criteria. Of these, 184 (50.1%) expressed a desire to have children, 96 (26.2%) reported not wanting children, and 87 (23.7%) indicated uncertainty about their parenthood desires. In accordance with the administered survey’s routing, only participants answering “Yes” to parenthood desire received the subsequent intention and geographic-preference items. The analytic sample therefore comprised 184 participants, 113 heterosexual and 71 LGB adults. This restriction resulted from planned routing rather than *post hoc* case selection.

### Instruments

Data for this study were collected through an online survey using Microsoft Forms. The following sections were analyzed in this manuscript:

#### Sociodemographic data

The first section of the survey collected sociodemographic information using predominantly single-choice categorical items. Participants reported their self-identified gender, sexual orientation, area of residence, occupational status, monthly income, highest educational level, religious affiliation and frequency of religious practice. Participants also had to identify their relationship status.

#### Parenthood aspirations

Parenthood desire was assessed using the item, “Would you like to be a father/mother in the future?.” Participants selected one of the three response options: “Yes,” “No,” and “I do not know.” The questionnaire used programmed routing based on this response. Participants selecting “Yes” continued to the parenthood-intention item and the subsequent parenthood module. Those selecting “No” were directed to a question about their reasons for not desiring parenthood, whereas those selecting “I do not know” exited the questionnaire and received a thank-you message. Consequently, the analyses of parenthood intentions and parenthood-related geographic preferences were restricted by design to participants who had expressed a desire for parenthood.

Among participants who selected “Yes,” parenthood intention was assessed with the follow-up item, “Do you have concrete plans to become a parent in the future?.” Response options were “Yes” or “No.” This item was treated as an indicator of stated parenthood intention rather than realized behavior. The parenthood desire and intention items were adapted from Baiocco and Laghi (2013). Desire and intention were treated as conceptually distinct constructs. Desire referred to wanting children, whereas intention referred to reporting concrete plans to pursue parenthood.

#### Parenthood-related geographic preferences

Geographic preference in relation to future parenthood was assessed using a study-specific item introduced by the following item: “How important would the following conditions be for you when deciding to become a parent and have children?” (“*Qué tan importantes serían para ti las siguientes condiciones al momento de decidir convertirte en padre/madre y tener hijos/as*?,” in Spanish). Participants were then asked to select between two geographic conditions, “Living in my current country of residence” and “Living in another country.”

Although the item stem referred to the importance of these conditions, the administered response format required participants to select one of two geographic alternatives and did not include a graded importance scale. Accordingly, the item was operationalized as a binary parenthood-geographic preference, coded as preference for the current country of residence (i.e., Ecuador), or preference for another country. It was not interpreted as a measure of the strength of the importance.

After selecting a geographic preference, participants provided an open-ended explanation. Those preferring their current country of residence were asked to explain why they wished to remain there. Those preferring another country were asked to explain their reasons and identify their preferred country or countries. The open-ended responses were used in the qualitative content analysis, while destination responses were summarized descriptively.

It should be noted that this measure was designed to capture the geographic setting participants considered preferable when imagining future parenthood. It does not assess migration timing, commitment, preparation, resources, legal feasibility, or subsequent relocation. Therefore, the construct is described as a parenthood-related geographic preference rather than migration intention, migration plan, or migration behavior. A preference for another country may reflect anticipated social, legal, economic, or family conditions, but the item does not independently determine which of these conditions produced the preference.

### Procedures

Data were collected between November 2022 and January 2023 through an anonymous online survey. Participants were recruited using a nonprobability strategy combining convenience and voluntary chain-referral sampling. Recruitment materials the survey link were distributed through Instagram, Facebook, and X (formerly known as Twitter); the professional networks of the research team; and the social-media accounts of the School of Psychology and Education at Universidad de Las Américas. Two organizations serving LGBTIQ+ communities also assisted with survey dissemination. Respondents were encouraged to voluntarily share and promote the survey within their social networks, an approach that has been effective in studies on minority and vulnerable populations (Ellard-Gray et al., 2015). Because recruitment chains were not formally tracked and recruitment probabilities were unknown, the procedure is more accurately described as online convenience and chain-referral sampling than as a probability-based or respondent-driven sampling.

The first survey page presented the informed-consent form. Participants were required to read and agree to the terms before completing the survey. The consent clearly explained the study’s purpose, participation conditions, and information regarding potential risks and benefits. Participation was voluntary and anonymous. Participants could decline participation by leaving the survey and could discontinue at any time by closing the browser window. Participants were also permitted to omit questions they did not wish to answer, except when a response was required to determine the appropriate survey route. No financial compensation was provided.

After providing consent, participants completed the sociodemographic section and the parenthood-desire item. The questionnaire then used programmed routing. Participants who answered “Yes” to the question “Would you like to be a father/mother in the future?” continued to the parenthood-intention item and the remaining parenthood module, including the parenthood-related geographic-preference questions. Participants who answered “No” were directed to a question asking about their reasons for not desiring parenthood. Those who selected “I do not know” did not continue to either branch and were shown a thank-you message. Consequently, administration of the intention and geographic-preference measures was restricted by design to respondents who had expressed a desire for future parenthood; this was not an exclusion introduced after data collection.

Within the parenthood module, participants selected whether they preferred to live in their current country of residence or another country when considering future parenthood. The selected response determined the subsequent open-ended prompt. Participants preferring their current country were asked to explain their reasons for remaining, whereas those preferring another country were asked to explain their reasons and identify their preferred destination or destinations. This routing allowed the quantitative geographic-preference response to be linked with the corresponding qualitative explanation.

The survey did not collect names, IP addresses, or email addresses, or other direct identifiers, and the survey platform was configured not to track respondents. Data was stored and analyzed in deidentified form. The study’s protocol was reviewed and approved by the Ethics Review Board of the Pontificia Universidad Católica del Ecuador (approval no. PV-20-2022). The study was considered to involve no more than minimal risk to participants.

### Data analysis

#### Quantitative analyses

Before analysis, the dataset was screened for coding inconsistencies, out-of-range values, and missing data. Categorical variables were examined using frequency distributions, and age was inspected using descriptive statistics and graphical methods. No observations were excluded solely based on their age values. Missingness was identified for age in five participants, educational attainment in one participant, and monthly income in one participant. Because these missing values occurred in different participants, analyses involving the complete set of covariates were based on 177 complete cases. Descriptive and bivariate analyses used all participants with valid data for the variables included in each analysis.

Participant characteristics were summarized using counts and percentages for categorical variables and the mean, standard deviation, median, and interquartile range for age. Sexual-orientation groups were compared descriptively across sociodemographic characteristics. The choice of test for each contingency table followed a prespecified rule based on expected cell frequencies. For 2 × 2 tables Pearson’s chi-square test with Yates’s continuity correction was used. For tables with more than two categories per variable the uncorrected Pearson’s chi-square test was used. Whenever any expected frequency fell below five, Fisher’s exact test was used instead. Cramér’s V was reported as an effect-size measure for categorical comparisons, accompanied by bootstrap 95% confidence intervals. Because the distribution of age did not meet the assumptions of normality, the Mann–Whitney U test was used to compare age distributions between the heterosexual and LGB groups, with the rank-biserial correlation and its 95% confidence interval reported as the accompanying effect size. These comparisons were used to describe the sample and identify group differences; they were not interpreted as demonstrating that the groups were statistically equivalent.

The primary quantitative outcome was parenthood-related geographic preference. Participants who selected “Living in another country” were coded as 1, and those selecting “Living in my current country of residence” were coded as 0. The association between sexual orientation and geographic preference was first examined using a 2 × 2 contingency table. Pearson’s chi-square test with Yates’s continuity correction was used for the primary bivariate significance test. The uncorrected Cramér’s V, unadjusted odds ratio, and corresponding 95% confidence interval were also reported to describe the magnitude and precision of the association.

A binary logistic-regression model was then estimated to examine whether sexual orientation was associated with preferring another country after adjustment for age, gender, educational attainment, employment status, and monthly income. The LGB group served as the reference category for sexual orientation, and preference for another country was modeled as the event. Woman, secondary education, working, and the lowest monthly-income category served as the reference categories for the remaining categorical predictors. All reference categories are identified in the regression table.

The regression was estimated using complete-case analysis (n = 177). Results are reported as unstandardized logit coefficients (B), standard errors, adjusted odds ratios, 95% confidence intervals, and two-sided *p* values. Overall model performance was evaluated using the likelihood-ratio test, Akaike information criterion, and McFadden’s pseudo-R^2^, and discrimination was summarized using the area under the receiver operating characteristic curve with a DeLong 95% confidence interval. A grouped goodness-of-fit assessment (Hosmer–Lemeshow) was used to evaluate calibration, and variance inflation factors were examined to identify potential multicollinearity; for the polytomous predictors, adjusted generalized variance inflation factors were obtained and influential observations were screened using Cook’s distance. Because the model included 14 predictor degrees of freedom with 68 participants selecting another country, estimates for categories containing few observations, particularly the highest-income category, were interpreted cautiously.

As a sensitivity analysis, a more parsimonious model containing sexual orientation, age, and gender was estimated to assess whether the orientation association was robust to a model with fewer parameters. The full adjusted model remained the primary analysis because it included the sociodemographic variables selected for adjustment, whereas the parsimonious model was used only to evaluate the stability of the principal association. Statistical significance was evaluated using a two-sided *α* level of 0.05. These analyses were conducted using the statistical software R 4.5.1 (R Core Team, 2025).

#### Qualitative analyses

Open-ended explanations of participants’ geographic preferences were examined using content analysis, focused primarily on manifest content, that is, the reasons explicitly expressed in each response (Vaismoradi et al., 2013). This approach was selected because the dataset consisted mainly of short survey responses and the objective was to identify and summarize recurring reasons rather than develop an interpretative account of latent meaning.

The two preference branches were analyzed separately. The first included explanations from participants who preferred their current country of residence; the second included explanations from participants who preferred another country. The individual participant response was the unit of analysis. Responses were first classified as interpretable, insufficiently elaborated, not applicable, or missing. Only interpretable responses were included when calculating the distribution of substantive content categories. Missing responses and statements that did not provide a discernible reason were documented separately and were not treated as substantive categories. In particular, “undisclosed” or “no stated reasoning” was not retained as a theme because it represented insufficient information rather than a reason for geographic preference.

The coding process proceeded iteratively. First, members of the research team read the responses to become familiar with their content and the range of reasons expressed. One researcher (PH-A) then conducted the initial coding by assigning concise descriptive codes to meaningful units within each response. No *a priori* codebook was imposed. Instead, codes were developed inductively from the data and were progressively compared, refined, and grouped into broader content categories. Category definitions, inclusion and exclusion criteria, and coding decisions were documented in a coding matrix that retained the connection between each original response and its assigned code or codes.

A single response could be assigned to more than one category when it contained multiple distinguishable reasons. For example, a response referring to both employment opportunities and personal safety could contribute to both categories. Consequently, categories were not mutually exclusive, and the sum of category percentages could exceed 100%. Category frequencies represented the number of participants whose responses were assigned to each category, rather than the total number of individual code applications.

After the initial coding and category development, a second researcher (CH-B) reviewed the coding matrix, the category structure, and the correspondence between responses and assigned codes. Discrepancies and ambiguous cases were discussed by the research team, and final assignments and category definitions were determined through consensus. This secondary review and consensus refinement is not an inter-rater reliability assessment and did not allow us to determine a reliability coefficient.

Category frequencies and percentages were calculated separately for heterosexual and LGB participants within each geographic-preference branch. The denominators were the numbers of participants in each group who provided interpretable responses in that branch. For current-country explanations, the denominators were 75 heterosexual and 28 LGB participants, for a total of 103 interpretable responses. For another-country explanations, the denominators were 30 heterosexual and 30 LGB participants, for a total of 60 interpretable responses. These percentages were used descriptively and were not subjected to inferential significance testing.

Preferred destination responses were summarized separately using frequencies because these entries generally consisted of country or regional names rather than explanations suitable for qualitative coding. Where participants could identify more than one destination, the analysis counted each named destination and explicitly noted that destination percentages might exceed 100%. Destination preferences were interpreted as stated preferences, not as evidence of migration preparation, legal feasibility, or subsequent relocation.

## Results

### Sample characteristics

The analytic sample comprised 184 childless cisgender adults residing in Ecuador who had expressed the desire for future parenthood. Of these participants, 113 (61.4%) identified as heterosexual and 71 (38.6%) as lesbian, gay, or bisexual (47 gay or lesbian and 24 bisexual participants). The sample included 111 women (60.3%) and 73 men (39.7%).

Age was available for 179 participants. The mean age was 27.32 years (SD = 6.02), and the median was 26 years (IQR = 23–31). Heterosexual participants were younger than LGB participants; heterosexual M = 25.79, SD = 5.11, median = 24; LGB M = 29.77, SD = 6.56, median = 29. The age distributions differed between groups, Mann–Whitney U = 2,313.50, *p* < 0.001, rank-biserial r = −0.39, 95% CI [−0.53, −0.23].

Descriptive group differences were also observed for gender, *χ*^2^(1) = 25.56, p < 0.001, Cramér’s *V* = 0.38, 95% CI [0.26, 1.00]; educational attainment, Fisher’s exact test, *p* = 0.036, *V* = 0.22, 95% CI [0.04, 1.00]; employment status, *χ*^2^(3) = 9.36, *p* = 0.025, *V* = 0.23, 95% CI [0.06, 1.00]; monthly income, Fisher’s exact test, *p* = 0.001, *V* = 0.33, 95% CI [0.16, 1.00]; and relationship status was comparable across groups, *χ*^2^(2) = 0.01, *p* = 0.993, *V* = 0.01, 95% CI [0.00, 1.00] (Table 1).

**Table 1:** Sociodemographic characteristics by sexual orientation.

Characteristic	Heterosexual	LGB	Total	*p*	Effect size
Age, years					
Mean (SD)	25.79 (5.11)	29.77 (6.56)	27.32 (6.02)		
Median [IQR]	24.00 [22.00, 28.75]	29.00 [24.00, 33.00]	26.00 [23.00, 31.00]	<0.001	r = −0.39 [−0.53, −0.23]
Gender, *n* (%)				<0.001	*V* = 0.38 [0.26, 1.00]
Woman	85 (75.2)	26 (36.6)	111 (60.3)		
Man	28 (24.8)	45 (63.4)	73 (39.7)		
Educational attainment, *n* (%)				0.036	*V* = 0.22 [0.04, 1.00]
Secondary	31 (27.4)	14 (20.0)	45 (24.6)		
Technical	5 (4.4)	7 (10.0)	12 (6.6)		
Undergraduate	58 (51.3)	27 (38.6)	85 (46.4)		
Master’s or doctoral	19 (16.8)	22 (31.4)	41 (22.4)		
Employment status, *n* (%)				0.025	*V* = 0.23 [0.06, 1.00]
Working	49 (43.4)	37 (52.1)	86 (46.7)		
Studying	30 (26.5)	6 (8.5)	36 (19.6)		
Studying and working	26 (23.0)	20 (28.2)	46 (25.0)		
Neither studying nor working	8 (7.1)	8 (11.3)	16 (8.7)		
Monthly income, US$, *n* (%)				0.001	*V* = 0.33 [0.16, 1.00]
0–450	57 (50.9)	17 (23.9)	74 (40.4)		
451–900	18 (16.1)	19 (26.8)	37 (20.2)		
901–1,300	17 (15.2)	8 (11.3)	25 (13.7)		
1,301–2,100	12 (10.7)	11 (15.5)	23 (12.6)		
2,101–4,220	7 (6.2)	13 (18.3)	20 (10.9)		
>4,220	1 (0.9)	3 (4.2)	4 (2.2)		
Relationship status, *n* (%)				0.993	*V* = 0.01 [0.00, 1.00]
Single	42 (37.2)	27 (38.0)	69 (37.5)		
Partnered, living apart	45 (39.8)	28 (39.4)	73 (39.7)		
Partnered, cohabiting	26 (23.0)	16 (22.5)	42 (22.8)		

Values are *n* (%) unless otherwise indicated. Percentages use valid data within each orientation group. Age: heterosexual *n* = 110, LGB *n* = 69, total *n* = 179. Educational attainment: heterosexual *n* = 113, LGB *n* = 70, total *n* = 183. Income: heterosexual *n* = 112, LGB *n* = 71, total *n* = 183. Gender, relationship status and employment: *N* = 184. Age was compared using a Mann–Whitney *U* test; categorical variables were compared using Pearson chi-square tests (with Yates’s continuity correction for 2 × 2 tables) or Fisher’s exact test when any expected frequency was < 5. Effect sizes are rank-biserial correlation for age and Cramér’s V for categorical variables, each with a bootstrap 95% confidence interval. LGB, lesbian, gay, or bisexual; SD, standard deviation; IQR, interquartile range.

*Research Question 1:* Was sexual orientation associated with parenthood-related geographic preference?

Overall, 115 of 184 participants (62.5%) preferred to live in their current country of residence when considering future parenthood, whereas 69 (37.5%) preferred another country. Among heterosexual participants, 81 of 113 (71.7%) preferred their current country and 32 (28.3%) preferred another country. Among LGB participants, 34 of 71 (47.9%) preferred their current country and 37 (52.1%) preferred another country (Table 2).

**Table 2:** Parenthood-related geographic preference by sexual orientation.

Geographic preference	Heterosexual, *n* (%)	LGB, *n* (%)	Total, *n* (%)
Current country of residence	81 (71.7)	34 (47.9)	115 (62.5)
Another country	32 (28.3)	37 (52.1)	69 (37.5)
Total	113 (100.0)	71 (100.0)	184 (100.0)

Percentages are calculated within sexual-orientation group. LGB = lesbian, gay, or bisexual.

The association between sexual orientation and geographic preference was statistically significant, Yates-corrected *χ*^2^(1, N = 184) = 9.54, *p* = 0.002. The uncorrected effect-size estimate was Cramér’s *V* = 0.24, 95% CI [0.12, 1.00]. In the unadjusted analysis, the odds of preferring another country were 2.75 times higher among LGB participants than among heterosexual participants, OR = 2.75, 95% CI [1.48, 5.12]. These results indicate an association with stated geographic preference.

*Research Question 2:* Did the association remain after sociodemographic adjustment?

A binary logistic-regression model examined whether sexual orientation remained associated with preferring another country after adjustment for age, gender, educational attainment, employment status, and monthly income. Preference for another country was modeled as the event. The reference categories were LGB identity, woman, secondary education, working, and monthly income of US$0–450.

The complete-case model included 177 participants, of whom 68 preferred another country; one participant who preferred another country was excluded from the model because of missing covariate data, which accounts for the difference from the 69 observed in the bivariate analysis. The overall model improved on an intercept-only model, likelihood-ratio *χ*^2^(14) = 24.72, *p* = 0.037. McFadden’s pseudo-R^2^ was 0.105, and AIC was 241.07. A grouped goodness-of-fit assessment did not indicate poor calibration, *p* = 0.857. Discrimination was acceptable, AUC = 0.72, 95% CI [0.64, 0.80] (DeLong). Multicollinearity was negligible, adjusted generalized variance inflation factors ranged from 1.14 and 1.71, all well below conventional thresholds. Influence diagnostics identified no unduly influential observations (maximum Cook’s distance = 0.139). Variance inflation factors were below 3.5. Table 3 contains the values for each variable.

**Table 3:** Logistic regression predicting preference for another country.

Predictor	*B*	*SE*	aOR	95% CI for aOR	*p*
Heterosexual vs. LGB	−1.35	0.40	0.26	[0.12, 0.56]	<0.001
Age, years	−0.08	0.05	0.92	[0.84, 1.02]	0.105
Man vs. woman	−0.03	0.42	0.97	[0.43, 2.21]	0.945
Technical vs. secondary education	−0.45	0.77	0.64	[0.14, 2.92]	0.564
Undergraduate vs. secondary education	−0.03	0.51	0.97	[0.36, 2.66]	0.956
Master’s/doctoral vs. secondary education	0.29	0.75	1.33	[0.31, 5.83]	0.701
Studying vs. working	0.13	0.61	1.13	[0.34, 3.78]	0.838
Studying and working vs. working	−0.26	0.49	0.77	[0.30, 2.00]	0.597
Neither studying nor working vs. working	0.39	0.68	1.48	[0.39, 5.59]	0.561
Income US$451–900 vs. 0–450	−0.63	0.55	0.53	[0.18, 1.59]	0.260
Income US$901–1,300 vs. 0–450	−0.76	0.69	0.47	[0.12, 1.82]	0.274
Income US$1,301–2,100 vs. 0–450	0.53	0.65	1.70	[0.47, 6.08]	0.415
Income US$2,101–4,220 vs. 0–450	0.13	0.81	1.14	[0.23, 5.60]	0.870
Income >US$4,220 vs. 0–450	1.55	1.43	4.73	[0.28, 78.58]	0.278

*n* = 177 complete cases, including 68 participants who preferred another country. The modeled event was preference for another country. aOR, adjusted odds ratio; CI, confidence interval; LGB, lesbian, gay, or bisexual. Reference categories were LGB, woman, secondary education, working, and monthly income US$0–450. Overall likelihood-ratio *χ*^2^(14) = 24.72, *p* = 0.037; McFadden’s *R*^2^ = 0.105; AIC = 241.07; AUC = 0.72, 95% CI [0.64, 0.80]; Hosmer—Lemeshow *p* = 0.857; maximum Cook’s distance = 0.139. Sparse-category estimates, particularly the highest-income category, should be interpreted cautiously.

After adjustment, heterosexual participants had lower odds of preferring another country than LGB participants, B = −1.35, SE = 0.40, aOR = 0.26, 95% CI [0.12, 0.56], *p* < 0.001. Age was not independently associated with geographic preference, B = −0.078, SE = 0.048, aOR = 0.92, 95% CI [0.84, 1.02], *p* = 0.105. Gender, education, employment, and income also showed no clear independent associations.

A parsimonious model containing sexual orientation, age, and gender produced a similar orientation estimate. Heterosexual participants had lower adjusted odds of preferring another country than LGB participants, aOR = 0.30, 95% CI [0.15, 0.62], *p* = 0.001. Age remained nonsignificant, aOR = 0.95, 95% CI [0.89, 1.00], *p* = 0.071, as did gender, aOR = 1.14, 95% CI [0.55, 2.37], *p* = 0.716.

*Research Question 3:* Why did participants prefer their current country or another country, and which destinations did they identify?

#### Qualitative response flow

Of the 115 participants who preferred their current country, 108 provided text. Five responses did not contain an interpretable reason, leaving 103 responses for substantive content analysis: 75 heterosexual and 28 LGB responses. Of the 69 participants who preferred another country, 68 provided text. Eight responses (one heterosexual and seven LGB) were insufficiently elaborated, leaving 60 interpretable responses, with 30 from each group. Blank and insufficient responses were documented separately and were not treated as substantive content categories. Table 4 provides information on the qualitative response flow.

**Table 4:** Qualitative response flow by geographic preference and sexual orientation.

Response status	Heterosexual, *n*	LGB, *n*	Total, *N*
Preferred current country			
Selected current country	81	34	115
Provided text	78	30	108
Interpretable response	75	28	103
Noninterpretable or insufficient text	3	2	5
Blank response	3	4	7
Preferred another country			
Selected another country	32	37	69
Provided text	31	37	68
Interpretable response	30	30	60
Noninterpretable or insufficient text	1	7	8
Blank response	1	0	1

“Provided text” includes responses subsequently judged insufficient for substantive coding.

#### Reasons for preferring current country

As shown in Table 5, family proximity and social support were the most frequently identified reasons for preferring the current country, appearing in 51 heterosexual responses (68.0%) and 13 LGB responses (46.4%), or 64 responses overall (62.1%). Participants described emotional proximity, assistance from relatives, and raising children near extended family. For many, particularly heterosexual individuals, the presence and accessibility of social support networks were the most influential factors driving this desire. For example, one respondent said, *“Because I feel that my children could be in greater contact with my family and even receive help from my parents in certain situations”* (Heterosexual woman, 23 years old). Another emphasized the role of extended family, stating, “*Because the support network, that is, the grandparents and the rest of the family, are in the country, and I would like them to be part of their growth and development”* (Heterosexual woman, 25 years old). One LGB participant wanted their children to be “close to their family and roots.”

**Table 5:** Reasons for preferring the current country of residence.

Content category	Examples	Heterosexual, *n* (%)	LGB, *n* (%)	Total, *n* (%)
Family proximity and social support	To have the company and support of my family. Because I feel that my children could have more contact with my family and even receive help from my parents in certain situations.	51 (68.0)	13 (46.4)	64 (62.1)
Cultural roots or identification with the country	I would like my child to know their country and the great cultures that exist within it. Because it would be important to know the customs and roots, to uphold identity so they grow with values, principles, and a sense of identity.	13 (17.3)	4 (14.3)	17 (16.5)
Employment, financial, or general stability	Ecuador is a country where, if you have the necessary economic conditions, you can live peacefully and comfortably. Because I have businesses and a stable life here.	11 (14.7)	5 (17.9)	16 (15.5)
Flexibility or indifference regarding location	I would like to live in Ecuador, but I could also live in another country without a problem.	7 (9.3)	4 (14.3)	11 (10.7)
Familiarity, practical ease, legal considerations, or established plans	I would prefer to raise my children in a country where I already have a relative understanding of how things work; I would not want to experiment with what parenthood would be like elsewhere. However, if financial circumstances were better abroad, I would not hesitate. My life plans are here unless something extraordinary happens.	5 (6.7)	5 (17.9)	10 (9.7)

Denominators are participants providing interpretable responses: heterosexual *n* = 75, LGB *n* = 28, total *N* = 103. Multiple codes were permitted; percentages may exceed 100%.

Cultural roots or identification with Ecuador appeared in 13 heterosexual responses (17.3%) and four LGB responses (14.3%). One participant noted, “*I would like them to learn about their parents’ culture and be involved with the family as I was in my time*” (Heterosexual woman, 21 years old). This connection to culture was also linked with tradition and well-being, as expressed by another participant, “*So that children can grow up with our customs and healthy food, as we are producers*” (Heterosexual, man, 20 years old). An older participant provided a broader perspective on cultural identity, highlighting values and principles, “*Because it would be important to know traditions, roots, and maintain identity so that it develops into values, principles, and identity*” (Gay man, 49 years old).

Stability appeared in 11 heterosexual responses (14.7%) and five LGB responses (17.9%). One respondent expressed this sentiment succinctly: “*Because financial stability is important*” (Gay man, 40 years old). Another participant, who had already built a life in Ecuador, explained, “*Because I have businesses and a stable life here*” (Gay man, 29 years old). Additionally, a participant reflected on both opportunity and social change, stating, “*I love my country, I have great opportunities here, and I believe the world is changing, and we must accept everyone*” (Gay man, 34 years old).

Flexibility or indifference regarding location appeared in seven heterosexual responses (9.3%) and four LGB responses (14.3%). Familiarity, practical ease, legal considerations, or established plans appeared in five responses from each group. One participant remarked, “*I believe it does not matter what country I am in. My child’s education will be the same anywhere in the world*” (Gay man, 30 years old), emphasizing that values and upbringing transcend geography. Similarly, another stated, “*Honestly, I would not have a problem with where I live as long as I am financially and emotionally stable. As long as my partner and I are well, that’s enough.*” (Heterosexual, woman, 25 years old), reinforcing the idea that stability, rather than location, is the defining factor in their decision-making process.

#### Reasons for preferring another country

Table 6 shows participants’ reasons for preferring another country. Better opportunities, quality of life, or perceived facilities abroad constituted the most common category in both groups. It appeared in 18 heterosexual and 18 LGB responses, representing 60.0% of each group. Participants referred to education, employment, social development, and opportunities for their future children. One participant expressed this sentiment clearly: “*I would like to have children living in another country since they can have a better quality of life and greater opportunities in life*” (Heterosexual woman, 24 years old). Similarly, another respondent stated, “*In the United States because there are more growth opportunities for future generations than in our countries*” (Gay man, 36 years old).

**Table 6:** Reasons for preferring another country.

Content category	Examples	Heterosexual, *n* (%)	LGB, *n* (%)	Total, *n* (%)
Better opportunities, quality of life, or facilities abroad	I would like to have children while living in another country since they could have a better quality of life and greater opportunities in life. In a country with a better quality of life, not just economically but also emotionally and socially. Some options: Spain, Portugal, Brazil.	18 (60.0)	18 (60.0)	36 (60.0)
Insecurity, poor quality of life, or limited local opportunities	Canada. The level of insecurity and quality of life in my current country is terrible. I would like to raise my children in a safe place where they can live well. I feel that Ecuador is becoming increasingly unstable, especially in terms of security and the economy, which are very important factors in raising a child.	7 (23.3)	2 (6.7)	9 (15.0)
Personal life plans, partner, or connections abroad	Germany. It is part of my life plan with my partner, and the quality of life there allows us to provide more opportunities for our children. Because of my partner’s nationality.	4 (13.3)	3 (10.0)	7 (11.7)
Restrictive social attitudes, discrimination, or rejection	In Ecuador it is very difficult for a gay couple to have children, and society is also very cruel to the children of gay couples. Ecuadorian society still has misguided stereotypes about what a family should be.	1 (3.3)	5 (16.7)	6 (10.0)
Cultural affinity with other countries	Brazil. Because I identify with its culture and plan to move there soon. I like Europe mainly because of its cultural development.	2 (6.7)	1 (3.3)	3 (5.0)
Flexibility or indifference regarding location	It really does not matter to me where I have and raise my children.	1 (3.3)	2 (6.7)	3 (5.0)

Denominators are participants providing interpretable responses: heterosexual *n* = 30, LGB *n* = 30, total *N* = 60. Multiple codes were permitted; percentages may exceed 100%. Frequencies are descriptive and were not subjected to significance tests.

Insecurity, poor quality of life, or limited opportunities in Ecuador appeared in seven heterosexual responses (23.3%) and two LGB responses (6.7%). One respondent emphasized, “*The level of insecurity and quality of life in my current country is terrible. I would like to raise my children in a safe place where they can live well*” (Heterosexual woman, 25 years old). Another participant added, “*I feel that Ecuador is becoming more unstable, especially in terms of security and the economy, which are crucial aspects for raising a child*” (Lesbian woman, 33 years old).

Personal plans, partners, or connections abroad appeared in four heterosexual responses (13.3%) and three LGB responses (10.0%). Restrictive social attitudes, discrimination, or anticipated rejection appeared in one heterosexual response (3.3%) and five LGB responses (16.7%). One participant pointed out, “*In Ecuador, it is very difficult for a gay couple to have children, and society is also very cruel to the children of gay parents*” (Gay man, 34 years old). Another echoed this concern, emphasizing that “*Ecuadorian society still has misguided stereotypes about what a family should be*” (Gay man, 35 years old).

Finally, some participants remained flexible and/or indifferent regarding the location where they would like to have and raise their children. As one participant put it, “*I honestly do not care where I have and raise my children*” (Gay man, 33 years old), reflecting a more adaptable stance toward migration decisions. It should be noted that these distributions are an interpretive qualitative pattern and therefore were not subjected to any inferential testing.

As shown in Table 6, the responses demonstrate that economic and quality-of-life considerations were common across sexual orientation groups. References to restrictive social attitudes, discrimination, and the anticipated treatment of same-gender-parent families appeared more often among LGB respondents.

#### Preferred destinations

**Figure 1:**
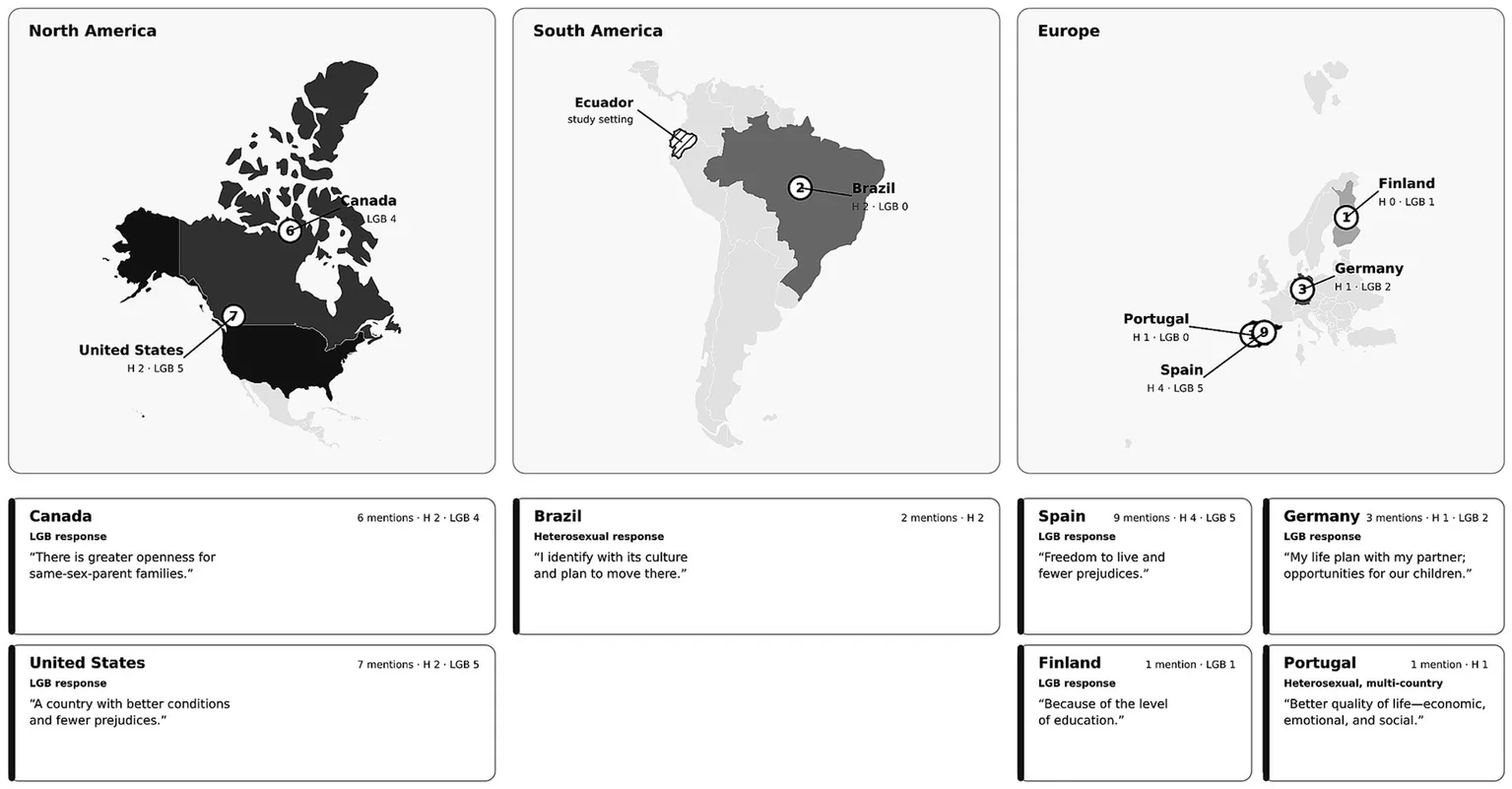
Parenthood-related preferred countries and illustrative responses. Quotes were translated from Spanish, deidentified, and lightly edited for spelling and clarity. Counts are destination mentions. Four respondents named only “Europe” and are not assigned to a country. The Portugal excerpt came from a response naming Spain, Portugal, and Brazil.

Among the 69 participants who preferred another country, 30 entered a location response. Twenty-six named at least one identifiable country, whereas four named only “Europe.” Because participants could identify multiple destinations, the 26 country-identifying respondents generated 29 country mentions. Spain was the most frequently named destination, with nine mentions, followed by the United States with seven and Canada with six. Germany received three mentions, Brazil two, and Portugal and Finland one each (see Figure 1).

Spain was associated with opportunities, cultural familiarity, quality of life, and fewer prejudices. The United States was associated mainly with opportunities for children’s development. Canada was connected to safety, legal conditions, and acceptance of same-gender-parent families. Germany was linked to quality of life and personal plans; Brazil to cultural identification and an existing relocation plan; Finland to education; and Portugal to a multi-country response emphasizing economic, emotional, and social quality of life.

## Discussion

This study aimed to explore how childless LGB and heterosexual adults residing in Ecuador who expressed a desire for parenthood evaluated the geographic context in which they might have and raise children. Focusing on childless individuals allowed us to capture anticipatory perspectives not yet shaped by the lived realities of raising children. This vantage point sheds light on how both LGB and heterosexual individuals envision opportunities and barriers to family formation within Ecuador’s sociocultural and legal context. Moreover, by integrating quantitative and qualitative data, we examined not only group differences in preferred geographic locations for having and raising children, but also the reasons associated with these preferences and the destinations mentioned by those who preferred living abroad.

By investigating these two dimensions together, this study offers a novel contribution to both research areas. Previous literature has explored LGB migration (e.g., Kalra and Bhugra, 2010; Yarwood et al., 2022; Yue, 2013) and LGB parenthood aspirations (e.g., Gato et al., 2019; Shenkman et al., 2021) as separate domains. Our findings suggest that these elements could be interconnected. The central quantitative finding was that LGB participants more frequently preferred living abroad when considering future parenthood than heterosexual participants. The bivariate association was moderate, and the association remained after adjustment for selected sociodemographic characteristics; heterosexual participants had lower adjusted odds of preferring another country than LGB participants. This result should not be interpreted as evidence that participants intended, planned, or were able to migrate. Rather, it indicates a difference in the geographic conditions under which future parenthood was imagined. Prospective parents may evaluate not only whether they want children, but also where they perceive family life to be feasible, secure, and socially supported. At the same time, the model accounted for a limited share of the variability in geographic preference, indicating that sexual orientation is one meaningful correlate among many rather than a dominant determinant.

The qualitative findings help contextualize this association. Across both groups, preferences were embedded in broader concerns about opportunity, quality of life, economic conditions, safety, and access to family support. These shared concerns are consistent with the current political, economic, and social conditions that have shaped evaluations of quality of life and mobility in Ecuador. They also illustrate the value of including a heterosexual comparison group as living abroad was not imagined exclusively in relation to sexual orientation, and many of the reasons participants provided reflected wider national conditions affecting prospective parents.

Family and emotional support were especially salient among those who preferred remaining in their current country of residence. Participants described proximity to relatives and established networks as resources for raising children. Furthermore, aspects related to cultural transmission and national identity were reported as important. More heterosexual participants emphasized the significance of raising children within their own culture and ensuring they learn about their country, culture, and language, a desire not as prominently expressed by LGB participants. Many individuals may see this as a legacy they wish to pass on to their children, achievable only by remaining in their home country.

At the same time, the open-ended responses suggested a sexuality-related layer in how some LGB participants evaluated place. Only LGB respondents explicitly referred to restrictive social attitudes, prejudice, or limited recognition of same-gender-parent families. Such accounts are consistent with literature describing sexual migration as shaped not only by efforts to escape violence or discrimination, but also by the pursuit of personal development, recognition, and conditions in which a valued life can be constructed (Bianchi et al., 2007; Kalra and Bhugra, 2010). In the context of prospective parenthood, perceived acceptance may concern both the individual and the anticipated treatment or legitimacy of the future family. However, these patterns must be further studied as the responses were brief and not every LGB participant referred to sexuality-related barriers.

These findings can be understood as reflecting unequal geographic opportunity structures surrounding family formation. Ecuador has made important advances in formal recognition, including marriage equality, yet formal equality does not necessarily translate into equivalent practical access to adoption, assisted reproduction, parental recognition, or family life free from discrimination. If some prospective parents perceive relocation as offering more favorable conditions for recognition, security, or acceptance, the effective exercise of family rights may become partly conditioned by the capacity to move. The study does not establish that migration is objectively necessary for LGB parenthood in Ecuador. It documents that a subset of participants perceived meaningful geographic differences in the conditions surrounding future family formation.

When exploring the countries to which people expressed their preferences, certain nations, such as Spain, the United States, and Canada, and European countries like Portugal, Germany, and Finland, were mentioned most frequently. In some cases, people did not specify a particular country but instead mentioned broader regions, such as Europe. These preferences likely reflect perceptions of these nations as economically stable and socially progressive, making them attractive locations for achieving better living conditions and creating supportive environments for creating life projects that align with their desires (Valenzuela, 2021). They should not be treated as evidence that these settings are uniformly inclusive or objectively superior. While these countries are often perceived as more inclusive and supportive of LGB families, recent political shifts, including the rise of populist governments with conservative agendas in the Global North, challenge these assumptions (Knauer, 2025). Policies and public discourse in some of these nations have increasingly restricted LGB rights, revealing a tension between perceptions of progressivism and the evolving sociopolitical landscape. In addition, recent evidence highlights the difficulties LGB individuals may encounter when arriving and integrating into life in countries such as Spain. Their expectations of host countries as safe havens may clash with the realities of struggling to meet basic needs, secure stable employment, and navigate subtle and overt discrimination within healthcare and judicial systems (Mahdipour et al., 2026). Future studies should therefore examine the trajectories of individuals who migrate to pursue parenthood, including their personal and family experiences throughout the migration and settlement process.

The socioeconomic implications are also important. International mobility requires financial resources, documentation, information, employment flexibility, and often the ability to leave established support networks. Migration may consequently be unattainable for participants with fewer resources, even when they perceive other countries as more favorable for family formation. Conversely, for some people experiencing scarcity, insecurity, or limited opportunity, remaining may not be perceived as viable, making migration appear to be the only conceivable route toward a better future. These two dynamics are different sides of the same structural problem: social and material inequalities shape both who can imagine relocation and who can realistically act on that preference. Prior work has similarly emphasized how class, gender, income, race, and sexual orientation intersect in the possibilities surrounding parenthood and mobility (Manalansan, 2006).

### Limitations

It is important to consider several limitations. One key limitation relates to the characteristics of the sample. In this study, LGB individuals differed significantly from heterosexual participants in factors such as age, gender, income level, occupation, and educational attainment. Relationship status, by contrast, was comparable across groups, which reduces the plausibility of partnership circumstances as an alternative explanation for the observed difference. Adjustment addressed selected differences in the quantitative model, but residual confounding remains possible. For instance, LGB individuals with higher levels of education and income may view international relocation as a viable life strategy for forming families, while those with fewer economic resources may perceive such paths as unattainable or remain excluded from them altogether. Similarly, the overrepresentation of younger heterosexual women in our sample may reflect generational or gendered pressures tied to caregiving or moral expectations around parenting. These intersecting axes of privilege and marginalization shape who can even contemplate parenthood through migration, and who may be silenced or discouraged from doing so. Future research should further explore these intersections, using analytical frameworks that account for how structural inequalities constrain or enable life-course trajectories.

A further limitation concerns the statistical precision of the adjusted model. The number of participants preferring another country was small relative to the number of predictors included, and some categories contained few observations. As a result, the estimates for individual sociodemographic characteristics are imprecise, and the model explained only a limited share of the variability in geographic preference. The association with sexual orientation was consistent across model specifications, but larger samples would be needed to estimate the contribution of each covariate with adequate precision.

In addition, the self-selection and recruitment of participants through social media may have further skewed the demographic characteristics of both LGB and heterosexual individuals. Social media-based recruitment tends to attract individuals who live in urban areas and are more digitally connected, potentially excluding those in rural areas with limited internet access, lower socioeconomic status, or different educational backgrounds (Sullivan et al., 2011). Thus, the recruitment methods in our study did not allow us to capture a sample that fully reflects the population’s diversity, particularly with respect to socioeconomic factors. Indeed, only a proportion of individuals have the economic means, job security, or social support to consider parenthood and migration as viable options. Those facing financial instability, precarious employment, or restrictive environments may perceive these life choices as unattainable or may not even contemplate them as possibilities. At the same time, for others in situations of scarcity, remaining in their current context may not be seen as a real option either, making migration appear as the only conceivable path forward. These two dynamics, migration as either unattainable or inevitable, illustrate different sides of the same coin in how socioeconomic conditions shape aspirations and perceived possibilities. Future studies should aim to recruit participants through a wider range of channels to ensure broader representation of experiences and desires related to family formation and migration. These channels could include outreach through community organizations, advocacy groups, and legal aid services that support LGB individuals and their families.

Another limitation lies in the questionnaire’s design and the questions’ phrasing. The question that assessed participants’ parenthood aspirations, including whether they would consider leaving the country to raise children, may not have captured the full complexity of their decision-making processes. In this study, we did not ask about the type of family they would like to create after migration, the methods by which they would prefer to have children, or other perceptions and beliefs about how migration may shape their parenthood aspirations. Also, the geographic outcome was assessed using a study-specific forced-choice item whose stem referred to the importance of living in the current country of residence or another country. This item did not distinguish an idealized geographic preference from migration desire, intention, planning, perceived feasibility, or subsequent behavior. The findings should therefore not be interpreted as evidence of actual migration decisions or strategies. Future research would benefit from more detailed, multi-item measures that separately capture the aspirational and practical dimensions of migration desires, as well as the perceived feasibility of migration in relation to parenting decisions. A more in-depth qualitative exploration of these issues is also important.

An additional limitation concerns our focus on only childless individuals who expressed a desire for parenthood. While this perspective is valuable for capturing anticipatory aspirations, future research would also benefit from comparing the views of those who already have children with those who do not. Such comparisons could provide richer insights into how lived experiences of parenting reshape migration preferences, perceptions of structural barriers, and evaluations of favorable family-raising contexts. Examining both groups could further clarify how aspirations evolve over time and across different stages of family life. In addition, our sample included only individuals living in Ecuador at the time of data collection, and we did not ask whether participants were Ecuadorian by birth, had naturalized, or had previously migrated to the country. Ecuador currently experiences significant inflows of migrants from countries such as Colombia and Venezuela (Jokisch, 2023), and individuals with diverse migration histories may have distinct aspirations and experiences regarding parenthood and family formation. Future research should consider including individuals with diverse migration histories and explicitly capture nationality to better understand how these factors intersect with parenthood intentions.

### Implications and future directions

Despite these limitations, this study provides valuable insights into how individuals evaluate geographic contexts in relation to their parenthood aspirations and why some may prefer to build their families outside their current country of residence. To the best of our knowledge, this study is among the first to explore this phenomenon, particularly within a context where research on LGB populations remains scarce. By examining the intersection of geographic preferences and family formation, the study advances the literature by shedding light on how shared national concerns, and, in some cases, sexuality-related factors shape decision-making processes in personal and family life. Furthermore, it highlights how these decisions interact with broader contextual factors such as perceived opportunities, social attitudes, legal frameworks, economic and security conditions. We hope this contribution opens new avenues for research on sexual migration and family formation, focusing on the role of geographic mobility as a perceived possibility for LGB individuals.

Moreover, the findings underscore the need for policies that recognize the complex ways in which LGB individuals build families, including the role migration plays in this process. While prior studies have established that LGB individuals may migrate to escape discrimination and seek sexual freedom (Carrillo, 2004; Kalra and Bhugra, 2010; Manalansan, 2006), our study extends this understanding by suggesting that preferences for living abroad may reflect not only reactions to restrictive conditions but also anticipated access to improved living conditions, legal recognition, and social acceptance. These findings must be understood within Ecuador’s particular sociopolitical reality. While same-sex marriage was legalized in 2019 through a Constitutional Court ruling, many legal and institutional barriers to family formation for LGB individuals remain unaddressed. Socially, LGB families are still viewed with suspicion or hostility in many sectors, and conservative political forces, often influenced by religious institutions, have opposed reforms that would expand rights for sexual minorities. These institutional and cultural conditions likely contribute to participants’ preferences for living abroad, not only in search of safety or rights, but also to the possibility of building their families with dignity and recognition. While further research is needed on this topic, addressing these barriers would ensure that migration is a choice rather than a necessity, allowing LGB individuals to build their families with dignity, security, and legal recognition in the places they actively choose to call home.

## Conclusion

This study indicates that the geographic context in which future parenthood is imagined differs between LGB and heterosexual adults residing in Ecuador. Among childless participants who desired parenthood, LGB adults more frequently preferred living abroad, including after adjustment for selected sociodemographic characteristics. Open-ended responses situated this difference within shared concerns, especially opportunity, quality of life, safety, and family support, and sexuality-related concerns expressed by a subset of LGB respondents. These findings do not demonstrate migration behavior or establish that relocation is necessary for parenthood. Rather, they show that prospective family formation may be evaluated through unequal geographic, social, and material opportunity structures.

It is crucial to examine how shifting legal frameworks, evolving social attitudes, and fluctuating economic conditions influence parenthood-related geographic preferences among LGB individuals. This research opens new avenues for examining how these preferences are not only shaped by sexuality but also intersect with issues of gender, social class, and broader social inequalities. As such, future studies should build on this work to further investigate the complex and multifaceted motivations behind migration, particularly in countries where LGB rights remain limited. By expanding our understanding of these issues, we can better inform policies that promote equity and inclusivity for diverse family structures.

## Data Availability

The datasets are not publicly available because the study concerns potentially sensitive information about sexual orientation, parenthood aspirations, and migration preferences. Deidentified quantitative data supporting the conclusions of this article may be made available by the corresponding author upon reasonable request, subject to applicable ethical and data-protection requirements. Open-ended qualitative responses may be subject to additional access restrictions because they can retain a risk of participant re-identification even after direct identifiers have been removed. Requests to access the datasets should be directed to cahermosa@usfq.edu.ec.

## Footnotes

1 In this paper, we use the term Global South to refer to countries primarily located in Africa, Latin America, Asia, and Oceania that share certain historical, economic, and political trajectories, and continue to face multiple challenges stemming from global inequalities (Dados and Connell, 2012). While the term is not strictly geographical, it serves to denote nations that occupy marginalized positions within global power structures relative to those of the Global North. Following Kloß (2017), we recognize that the Global South is not a fixed or inherent category, but rather something that is “created, imagined, invented, maintained, and recreated by the ever-changing and never fixed status positions of social actors and institutions” (p. 1).
