# Supplementary material for: Envisioning family, but where? Parenthood-related geographic preferences among childless LGB and heterosexual adults in Ecuador
Source: Front Sociol. 2026 Aug 21;11:1876346. doi: 10.3389/fsoc.2026.1876346 (PMC13541527; doi:10.3389/fsoc.2026.1876346)
Supplement: Supplementary file 1 [file Table_1.DOCX]

**Supplementary Materials**

**Envisioning Family, But Where? Parenthood-Related Geographic Preferences among Childless LGB and Heterosexual Adults in Ecuador**

# Supplementary Table S1. Participant eligibility, routing, and analytic-sample construction

**Table S1. Participant eligibility, routing, and analytic-sample construction**

| **Stage** | **n** | **Operational decision** |
| --- | --- | --- |
| Initial online-survey participants | 561 | Entered the broader parenthood-aspirations study. |
| Met article-level demographic eligibility criteria | 367 | Adults residing in Ecuador; no biological or adoptive children; cisgender; sexual orientation coded as heterosexual, gay/lesbian, or bisexual. |
| Answered “Yes” to parenthood desire | 184 | Received/entered the follow-up module used in this article; retained as the analytic sample. |
| Answered “No” to parenthood desire | 96 | Not included because geographic-preference questions were conditional on desiring parenthood. |
| Answered “I do not know” | 87 | Not included; this response terminated progression into the analytic follow-up module. |
| Final heterosexual analytic group | 113 | Participants identifying as heterosexual. |
| Final LGB analytic group | 71 | 47 gay/lesbian and 24 bisexual participants combined for the primary comparison. |
| Geographic-preference analysis | 184 | Complete outcome data: 115 selected current country of residence; 69 selected another country. |
| Full adjusted regression | 177 | Complete cases after excluding five missing age values, one missing education value, and one missing income value; missing values occurred in different participants. |

**Note.** The analytic population is conditional: childless cisgender heterosexual and LGB adults residing in Ecuador who expressed a desire for future parenthood. The study does not estimate the prevalence of parenthood desire in the broader population.

# Supplementary Table S2. Survey items, response formats, routing, and analytic use

**Table S2. Survey items, response formats, routing, and analytic use**

| **Construct** | **Exact Spanish item** | **English translation** | **Response format** | **Routing** | **Analytic use** |
| --- | --- | --- | --- | --- | --- |
| Parenthood desire | ¿Te gustaría ser padre/madre en el futuro? | Would you like to be a father/mother in the future? | Yes; No; I do not know | Yes → follow-up module; No → reasons for not desiring parenthood; I do not know → module ended | Eligibility/routing variable. All N=184 included participants answered Yes. |
| Parenthood intention | ¿Tienes planes concretos de convertirte en padre/madre en el futuro? | Do you have concrete plans to become a parent in the future? | Yes; No | Displayed after Yes to Item 25 | Descriptive contextual variable; distinct from desire and from geographic preference. |
| Parenthood-related geographic preference | ¿Qué tan importantes serían para ti las siguientes condiciones al momento de decidir convertirte en padre/madre? | How important would the following conditions be for you when deciding to become a parent? | Estar viviendo en mi país de residencia actual (living in my current country of residence); Estar viviendo en otro país (living in another country) | Displayed in the parenthood follow-up module; administered as a single choice in the analytic data | Binary outcome: current country of residence=0; another country=1. |
| Current-country explanation | Si marcaste preferir vivir en tu país de residencia actual, indica el por qué de tu respuesta: | If you indicated that you preferred living in your current country of residence, explain why: | Open text | Displayed after selecting current country | Qualitative content analysis of interpretable responses. |
| Another-country destination and explanation | Si marcaste preferir vivir en otro país, indica cuál y el por qué de tu respuesta: | If you indicated that you preferred living in another country, specify which country and explain why: | Open text | Displayed after selecting another country | Qualitative content analysis plus descriptive count of country mentions. |

# Supplementary Table S3. Qualitative response flow

**Table S3. Qualitative response flow by geographic preference and sexual orientation**

| **Preference** | **Response status** | **Heterosexual, n** | **LGB, n** | **Total, N** |
| --- | --- | --- | --- | --- |
| Current country | Selected option | 81 | 34 | 115 |
| Current country | Provided text | 78 | 30 | 108 |
| Current country | Interpretable response | 75 | 28 | 103 |
| Current country | Insufficient/noninterpretable text | 3 | 2 | 5 |
| Current country | Blank response | 3 | 4 | 7 |
| Another country | Selected option | 32 | 37 | 69 |
| Another country | Provided text | 31 | 37 | 68 |
| Another country | Interpretable response | 30 | 30 | 60 |
| Another country | Insufficient/noninterpretable text | 1 | 7 | 8 |
| Another country | Blank response | 1 | 0 | 1 |

**Note.** “Provided text” includes responses later judged insufficient for substantive coding. Blank and insufficient responses were documented separately and were not treated as content categories. The denominators for category percentages were N=103 for current-country responses and N=60 for another-country responses.

# Supplementary Table S4. Final qualitative coding framework

**Table S4. Final codebook for interpretable open-ended responses**

| **Preference** | **Category** | **Operational definition** | **Illustrative translated excerpt** | **Total, N (%)** |
| --- | --- | --- | --- | --- |
| Current country | Family proximity and social support | References to relatives, friends, emotional proximity, practical help, or raising children near extended family. | “To have the company and support of my family.” | 64 (62.1%) |
| Current country | Cultural roots or identification with the country | Belonging to Ecuador; transmission of customs, language, roots, identity, or national culture. | “I would like my child to know their country and its cultures.” | 17 (16.5%) |
| Current country | Employment, financial, or general stability | Established employment, business, housing, finances, or a stable life locally. | “I have businesses and a stable life here.” | 16 (15.5%) |
| Current country | Flexibility or indifference regarding location | No strong locational preference; openness to either country if core conditions are met. | “I could live in another country without a problem.” | 11 (10.7%) |
| Current country | Familiarity, practical ease, legal considerations, or established plans | Knowledge of how the country works, perceived practical ease, local legal considerations, or life plans already located in Ecuador. | “My life plans are here unless something extraordinary happens.” | 10 (9.7%) |
| Another country | Better opportunities, quality of life, or facilities abroad | Perceived advantages abroad in education, employment, services, development, or general quality of life. | “They could have a better quality of life and greater opportunities.” | 36 (60.0%) |
| Another country | Insecurity, poor quality of life, or limited local opportunities | Crime, instability, economic deterioration, limited services, or lack of opportunity in Ecuador. | “Ecuador is becoming increasingly unstable, especially in security and the economy.” | 9 (15.0%) |
| Another country | Personal life plans, partner, or connections abroad | A partner’s nationality/location, family or social ties abroad, or an existing personal relocation trajectory. | “It is part of my life plan with my partner.” | 7 (11.7%) |
| Another country | Restrictive social attitudes, discrimination, or rejection | Prejudice, heteronormative family expectations, discrimination, or anticipated rejection of the participant, couple, or future child. | “Raising a child in Ecuador as a same-sex couple would not be easy.” | 6 (10.0%) |
| Another country | Flexibility or indifference regarding location | No strong preference despite selecting another country; location framed as secondary. | “It really does not matter where I have and raise my children.” | 3 (5.0%) |
| Another country | Cultural affinity with other countries | Identification with or attraction to another country/region’s culture. | “I identify with its culture.” | 3 (5.0%) |

**Note.** Denominators: current-country preference N=103; another-country preference N=60. Multiple codes were permitted, so percentages may exceed 100%. Excerpts were translated from Spanish, deidentified, and lightly edited for spelling and clarity. Insufficiently elaborated responses were excluded from substantive coding.

**Supplementary Table S5. Preferred destinations**

**Table S5. Country mentions among participants preferring another country**

| **Preferred country** | **Heterosexual mentions, n** | **LGB mentions, n** | **Total mentions, N** |
| --- | --- | --- | --- |
| Spain | 4 | 5 | 9 |
| United States | 2 | 5 | 7 |
| Canada | 2 | 4 | 6 |
| Germany | 1 | 2 | 3 |
| Brazil | 2 | 0 | 2 |
| Portugal | 1 | 0 | 1 |
| Finland | 0 | 1 | 1 |
| Total country mentions | 12 | 17 | 29 |

**Note.** Among 69 participants preferring another country, 30 entered a location response. Twenty-six named at least one identifiable country and generated 29 country mentions because multiple destinations were permitted. Four additional respondents entered only “Europe” and are excluded from this country-specific table. Mentions indicate stated preferences, not migration plans or feasibility.

# Supplementary Table S6. Parsimonious sensitivity model

**Table S6. Parsimonious logistic regression predicting preference for another country**

| **Predictor** | **aOR** | **95% CI** | **p** |
| --- | --- | --- | --- |
| Heterosexual vs. LGB | 0.30 | [0.15, 0.62] | .001 |
| Age, years | 0.95 | [0.89, 1.00] | .071 |
| Man vs. woman | 1.14 | [0.55, 2.37] | .716 |

**Note.** The modeled event was preference for another country. Reference categories were LGB and woman. This sensitivity model was specified to assess whether the sexual-orientation estimate remained similar under a lower-dimensional adjustment set. The association was substantively consistent with the fully adjusted model (main-manuscript Table 3). aOR=adjusted odds ratio; CI=confidence interval; LGB=lesbian, gay, or bisexual.

# Supplementary Table S7. Missing data and analysis-specific denominators

**Table S7. Missing data and analysis-specific denominators**

| **Variable/analysis** | **Valid denominator** | **Missing/excluded** | **Treatment** |
| --- | --- | --- | --- |
| Age | 179 | 5 | Descriptive comparison and adjusted regression |
| Gender | 184 | 0 | Descriptive comparison and adjusted regression |
| Educational attainment | 183 | 1 | Descriptive comparison and adjusted regression |
| Employment status | 184 | 0 | Descriptive comparison and adjusted regression |
| Monthly income | 183 | 1 | Descriptive comparison and adjusted regression |
| Geographic-preference outcome | 184 | 0 | Bivariate outcome analysis |
| Full adjusted regression | 177 complete cases | 7 participants excluded | Listwise complete-case analysis; missing age, education, and income occurred in different participants |
| Current-country qualitative categories | 103 interpretable responses | 12 of 115 not in substantive denominator | Seven blank and five insufficient responses |
| Another-country qualitative categories | 60 interpretable responses | 9 of 69 not in substantive denominator | One blank and eight insufficient responses |
| Country-specific destination counts | 26 participants; 29 mentions | 43 of 69 did not name an identifiable country | Four additional participants named only Europe |

**Note.** No imputation was performed. The bivariate geographic-preference analysis used all N=184 participants. The fully adjusted model used n=177 complete cases. Qualitative exclusions concern the adequacy of text for category assignment and were documented rather than recoded as substantive reasons.
